# A High-Luminescence Biomimetic Nanosensor Based on N, S-GQDs-Embedded Zinc-Based Metal–Organic Framework@Molecularly Imprinted Polymer for Sensitive Detection of Octopamine in Fermented Foods

**DOI:** 10.3390/foods11091348

**Published:** 2022-05-06

**Authors:** Ying Guo, Guanqing Yuan, Xuelian Hu, Jinni Zhang, Guozhen Fang

**Affiliations:** State Key Laboratory of Food Nutrition and Safety, Tianjin University of Science and Technology, Tianjin 300457, China; guoyingaxx@163.com (Y.G.); 15731366559@126.com (G.Y.); hxl523686206@126.com (X.H.); zjn939699@163.com (J.Z.)

**Keywords:** N/S co-doped graphene quantum dots, zeolitic imidazolate framework-8, molecularly imprinted polymer, octopamine, fluorescent sensor

## Abstract

In this study, a novel fluorescent molecularly imprinted nanosensor (N, S-GQDs@ZIF-8@MIP) based on the nitrogen and sulfur co-doped graphene quantum dots decorated zeolitic imidazolate framework-8 was constructed for the detection of octopamine (OA). Herein, ZIF-8 with a large surface area was introduced as a supporter of the sensing system, which effectively shortened the response time of the sensor. Meanwhile, high green luminescent N, S-GQDs and a maximum emission wavelength of 520 nm under 460 nm excitation and a 12.5% quantum yield were modified on the surface of ZIF-8 as a signal tag that can convert the interactions between the sensor and OA into detectable fluorescent signals. Finally, N, S-GQDs@ZIF-8@MIP was acquired through the surface molecular imprinting method. Due to the synergy of N, S-GQDs, ZIF-8, and MIP, the obtained sensor not only demonstrated higher selectivity and sensitivity than N, S-GQDs@ZIF-8@NIP, but also displayed faster fluorescence response than N, S-GQDs@MIP. Under optimal conditions, the developed sensor presented a favorable linear relationship in the range of 0.1–10 mg L^−1^ with a detection limit of 0.062 mg L^−1^. Additionally, the proposed N, S-GQDs@ZIF-8@MIP strategy was effectively applied to the detection of OA in fermented samples, and the obtained results had a satisfactory correlation with those of HPLC.

## 1. Introduction

Biogenic amines (BAs) are a type of biologically active containing nitrogen organic compound related to the physiological functions of humans [1]. Octopamine (OA) is a typical organic matter of BAs, named after its initial discovery in octopus saliva and has been known as a natural β3-adrenergic receptor agonist in the nervous system [2]. The intaking of excessive OA can cause toxic effects [3], such as headaches, vomiting, abnormal blood pressure, skin allergies, and other symptoms, and has led to brain hemorrhages in severe cases. In 2004, OA was banned in all sports competition since its stimulating properties may reduce the immunity of the organism and damage the sensory organs [4]. Therefore, as OA poses a potential public health threat, the detection of OA has become an important indicator of food safety. To date, various traditional analytical methods for the quantitative determination of OA have been developed, including electrochemical sensor detection [5], pseudo-ELISA [6], fast-scan cyclic voltammetry (FSCV) [7], LC-MS/MS method [8], and high-performance liquid chromatography (HPLC) [8]. Although these methods have made large progress for the detection of OA, when confronted with the detection of OA in fermented foods, these methods are limited by the need for laborious pretreatment, high costs, and interference by environment [9]. Therefore, it is particularly important to develop inexpensive and highly sensitive approaches for the detection of OA.

Graphene quantum dots (GQDs) have attracted widespread attention as a novel kind of nanomaterial due to their unique properties, such as distinctive electron transfer, strong chemical inertness, and low toxicity [10]. Recently, many studies have proved that the photoelectric characteristics of GQDs can be efficiently adjusted via doping heteroatoms [11]. Additionally, it was demonstrated that doped GQDs have excellent optical properties compared to non-doped GQDs [10,12]. The introduction of the N atom in the GQD structure helps to improve the quantum yield. Previous works have indicated that nitrogen-doped GQDs exhibit a higher quantum yield than non-doped GQDs [13]. S, as a heteroatom, is also widely used for GQD synthesis because it can effectively change the electron structure of the GQDs to produce multiple emission peaks by introducing S-related energy levels between the π and π* of C [14]. Li et al. have implied that S-GQDs had a sensitive reaction to Fe^3+^ because the introduction of a S atom improved the electronic properties and surface chemical reactivities [15]. After doping these GQDs, the N, S-GQDs exhibited excellent optical performance, including multiple emission peaks and high fluorescent quantum yield. For example, Qu et al. developed N, S-GQDs with three absorption bands independent of the excitation wavelength [16]. Thus, N, S-GQDs can be considered fluorescent materials with good application prospects.

Metal–organic frameworks (MOFs) composed of central metal ions and organic ligands are also known as porous coordination polymers (PCPs) [17]. Over the past few years, MOFs have drawn increasing attention in the research areas of gas adsorption and separation [18], drug delivery [19], electrochemical energy storage [20], and sensing [21] due to their special large surface areas, tunable porosity, and versatile architectures [22]. However, to date, their sensing functions of MOFs have lacked signal transduction capacity, which has severely restricted their development. Encapsulating luminescent molecules or nanoparticles into the non-luminescent MOFs could solve this problem [23]. Our previous research has revealed that embedding CDs as the fluorescence signal into the zeolitic imidazolate framework (ZIF-8) can acquire sensitizing materials [24]. Therefore, this strategy can not only obtain composite material with fluorescence-sensing properties but also improve the dispersion of luminescent molecules. Although the as-prepared composite material has good sensitivity, it still faces interference from other substances in the detection system due to the non-specificity of the fluorescent sensor.

In recent years, the use of molecular imprinting technology to modify MOFs has gradually aroused the interest of researchers. Molecular imprinting technology is a biomimetic technique based on the specific reaction between the antigen and antibody, which has been applied to prepare the polymers with specific structures [25]. The formed polymers are named molecularly imprinted polymers (MIPs) and not only exhibit selectivity for a target molecule or a group of structurally related analogies, but also have higher stability and longer service lives as well as tolerance to different environments [26]. Based on these excellent properties, MIPs are widely used to build sensing, separation, and catalytic platforms [27]. In the past few years, there have been a few studies on octopamine (OA) determination based on molecularly imprinted fluorescent nanoparticles [28]. Although the aforementioned studies determined that molecular imprinting technology can be used as a facile, reliable, and rapid strategy for OA detection via the formation of specific recognition sites, such methods frequently have low sensitivity and selectivity, limiting their applications in the field of optical sensing. Hence, it is of great significance to develop a fluorescent sensor with exceptional sensitivity and selectivity.

Inspired by this previous research, we designed and developed a novel fluorescent nanosensor N, S-GQDs@ZIF-8@MIP based on the N, S-GQDs-embedded zeolitic imidazolate framework-8 @ molecularly imprinted polymer. In this work, as the supporter, ZIF-8 with a large surface area was first prepared via the self-assembly method. Subsequently, N, S-GQDs-functionalized ZIF-8 (N, S-GQDs@ZIF-8) was synthesized by encapsulating N, S-GQDs using extremely bright-green light within ZIF-8 under convenient room temperature stirring. Finally, N, S-GQDs@ZIF-8@MIP with high selectivity and recognition ability for target molecules was fabricated through the sol–gel approach in a mixed solution containing N, S-GQDs (fluorophore), ZIF-8 (supporter), OA (template), 3-aminopropyl triethoxysilane (functional monomer), and tetraethyl orthosilicate (cross-linker). The incorporated bright-green luminescent N, S-GQDs, as signal bands, converts the chemical reaction with OA into detectable fluorescence signals, thereby realizing a highly sensitive response to OA. ZIF-8 with a high specific surface area, as a supporter, can improve the dispersion of N, S-GQDs and increase the mass transfer rate. Additionally, the imprinted layer decorated on the surface of the N, S-GQDs@ZIF-8 provided the specific recognition site for the target molecule. The uniform morphology, superior photostability, highly selective recognition performance, and ultra-sensitive fluorescence response of the as-obtained N, S-GQDs@ZIF-8@MIP were highly confirmed by the characterizations. The practicality of the proposed sensor was verified through realizing sensitive detection of OA in real samples. The preparation and characterization of the obtained N, S-GQDs@ZIF-8@MIP were discussed in detail.

## 2. Materials and Methods

### 2.1. Materials and Instruments

All the reagents were analytically pure and used as received. 2-Methylimidazole (2-MI, 99%) and *N*,*N*-Dimethylformamide (DMF) were acquired from TCI (Shanghai, China). 3-aminopropyl triethoxysilane (APTES, 98%), citric acid (CA, 99%), tetraethylorthosilicate (TEOS, 99%), zinc nitrate hexahydrate (Zn(NO_3_)_2_·6H_2_O, 99%), thiourea, glucose and sucrose were obtained from Aladdin Chemistry (Shanghai, China). KCl, MgCl_2_, NaCl, FeCl_3_, L-Arginine (L-Arg), D-Tryptophan (D-Try), L-Methionine (L-Met), L-Cysteine (L-Cys), L-Histidine (L-His), L-Tryptophan (L-Try), L-Tyrosine (L-Tyr), Dopamine (DA) and OA were supplied by Shanghai Macklin Biochemical Co., Ltd. (Shanghai, China). Na_2_HPO_4_⋅12H_2_O was purchased from Sinopharm Chemical Reagent Co., Ltd. (Tianjin, China). Information on the instruments was provided in the supporting information.

Fluorescence spectra were performed on an F-7100 fluorescence spectrophotometer (Thermo, Waltham, MA, USA) at the excitation wavelength of 460 nm. Fourier-transform infrared (FT-IR) was performed to analyze the obtained materials on a Tensor-37 FTIR spectrophotometer (Bruker, Bremen, Germany). Scanning electron microscope (SEM) and transmission electron microscope (TEM) images were used to show the surface and structure of the obtained materials (Waltham, MA, USA). Ultraviolet-visible (UV-Vis) absorption spectra were obtained on UV-Cary100 spectrophotometer (Shimadzu, Kyoto, Japan). Thermogravimetric analysis (TGA) was carried out with a PTC-10 A thermal gravimetric analyzer (Rigaku Corp., Tokyo, Japan) from room temperature to 900 °C. X-ray diffraction (XRD) data were measured at the angular range of 5–60 degrees (2θ) with Cu Kα radiation.

### 2.2. Synthesis of ZIF-8, N, S-GQDs and the N, S-GQDs@ZIF-8@MIP Composite

ZIF-8 was prepared as described in the previous literature via the one-pot synthesis method at room temperature [29]. In brief, 125 mg of Zn(NO_3_)_2_·6H_2_O and 275.9 mg of 2-MI were dispersed in 10 mL of methanol with sonicating for 30 min. Subsequently, the solutions were transferred to a 100 mL flask and mixed well under a magnetic stirrer. This reaction was maintained for about 50 min. The resulting products were centrifuged at 10,000 rpm for 5 min and dried at 50 °C under a vacuum for 8 h.

N, S-GQDs were synthesized via the solvothermal route based on the method reported previously [16]. Briefly, 1 mmol citric acid and 3 mmol thiourea were added into 3 mL *N*,*N*-Dimethylformamide with magnetic stirring for 30 min. The mixture was then moved to an autoclave and maintained at 180 °C for 8 h under N_2_ gas. The N, S-GQDs were collected from the suspension, centrifuged at 10,000 rpm for 10 min, and washed with ethanol. The precipitate was then dispersed into ethanol for subsequent use.

The surface-imprinted sol–gel technique was used to synthesize N, S-GQDs@ZIF-8@MIP. The product synthesis was achieved as follows. The as-prepared ZIF-8 and 200 μL N, S-GQDs were added into ethanol and stirred continuously until completely dispersed. Then, 1 mmol OA and 4 mmol APTES were dispersed into the solution with magnetic stirring for 30 min. Under continuous stirring, 6 mmol TEOS and ammonia water were added stepwise. After being pre-assembled, the mixed solution was sealed with a sealing film and stirred under a N_2_ atmosphere for 10 h. The final material was obtained through washing with ethanol until no template molecules of OA could be detected. N, S-GQDs@ZIF-8@NIP was acquired using an identical procedure, but no OA was added during preparation. N, S-GQDs@MIP was synthesized in the same manner while avoiding the addition of ZIF-8.

### 2.3. Fluorescence Measurement

Fluorescence (FL) measurements were accomplished under an excitation wavelength of 460 nm using a Hitachi FL-7000 fluorescence spectrophotometer (Tokyo, Japan). In total, 1.0 mg N, S-GQDs@ZIF-8@MIP, N, S-GQDs@ZIF-8@NIP, or N, S-GQDs@MIP was dispersed in different 2 mL OA concentration solutions at 20 ± 5 °C, under laboratory relative humidity of 50–70%. After about 40 min of incubation, a quantitative analysis was performed. The fluorescence intensity of the sensing system at 520 nm was tested under excitation of 460 nm. Selectivity was evaluated by adding OA or other potentially coexisting substances under the above experimental conditions.

### 2.4. Pretreatment of Samples

In order to evaluate the accuracy of the developed N, S-GQDs@ZIF-8@MIP sensor in detecting OA in samples, wine and white vinegar were taken as representative fermented samples. Additionally, we studied three spiked concentration (1, 5, 9 mg L^−1^) levels via spike recovery experiments. The samples were vortexed evenly and placed overnight. The procedure used to prepare the samples was as follows. In short, 4 mL of 5% trichloroacetic acid was mixed with 1 mL of the sample for 2 min. After centrifugation was performed at 3600 rpm for 10 min to collect the supernatant, 4 mL of n-hexane was added to the supernatant to remove the fat. Next, the extract’s pH was adjusted to 7.0 with an NaOH solution, and the extract was evaporated to dryness. Finally, the residue was redissolved with 1 mL of ultrapure water and filtered with a microporous membrane for FL detection. Then, for FL analysis, 1.0 mg N, S-GQDs@ZIF-8@MIP was placed in a 1 mL sample solution and incubated for 40 min. This method was verified via HPLC. The pretreatment method for wine and white vinegar was provided in the supporting information.

For HPLC detection, the samples that were extracted and defatted as abovementioned were derivatized with dansyl chloride and then analyzed according to the National Standards of the People’s Republic of China (GB 5009.208-2016). The analyses were performed using an HPLC system equipped with an ultraviolet detector. The HPLC operating parameters were as follows: The separation was carried out with a reversed phase column (C18, 250 × 4.6 mm, 5 μm particle size) with a flow rate of 0.8 mL min^−1^ and a column temperature of 35 °C. The injection volume was 20 μL, and the detection wavelength was 254 nm.

## 3. Results and Discussion

### 3.1. Preparation of N, S-GQDs, ZIF-8 and N, S-GQDs@ZIF-8@MIP

**Scheme 1:**
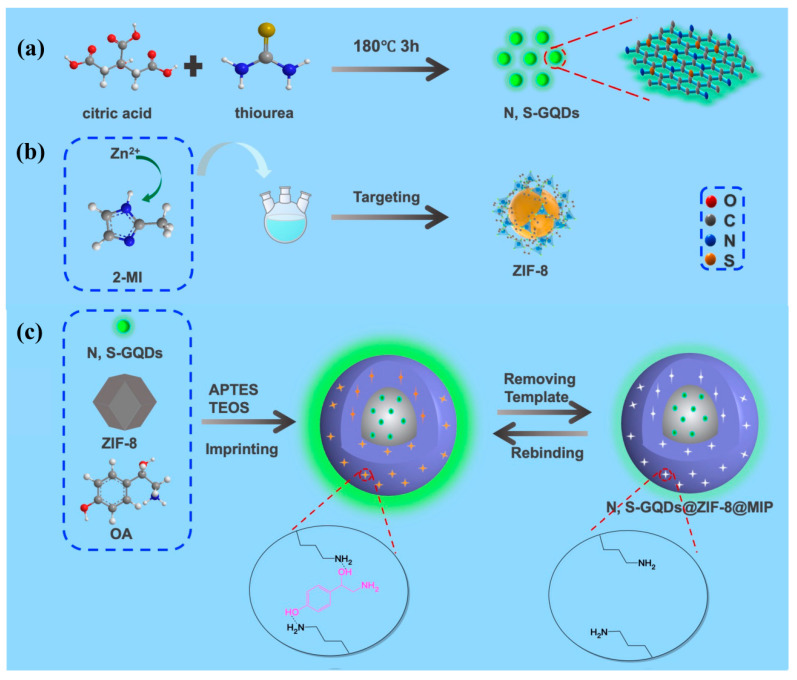
Preparation process of N, S-GQDs (**a**), ZIF-8 (**b**), and N, S-GQDs@ZIF-8@MIP sensor (**c**).

As displayed in Scheme 1, a novel synthetic strategy was proposed to prepare a fluorescent sensor constructed from MIP, N, S-GQDs, and ZIF-8 for the sensitive detection of OA. N, S-GQDs were synthesized through the solvothermal route based on citric acid as a carbon source and thiourea as nitrogen and sulfur sources (Scheme 1a). Compared to other GQDs, the obtained N, S-GQDs were excitation-dependent and showed different types of emission light under multiple excitation conditions [30]. It is worth noting that N, S-GQDs not only displayed extremely bright green light but also had a quantum yield (QY) as high as 12.5% [16]. The above phenomenon can be explained as follows. On the one hand, the high QY may be attributed to the doping of nitrogen atoms that changing the optical and electronic structure of the graphene quantum dots [31]. On the other hand, the green-fluorescent N, S-GQDs could benefit from the conjugation of the C=N, C=S, and C=O groups with the graphene core [32]. As presented in Scheme 1b, the high surface area ZIF-8 was prepared via the one-pot synthesis method. The introduction of ZIF-8 with a high specific surface area not only improved the dispersibility of GQDs, but also increased the mass transfer rate efficiently.

As illustrated in Scheme 1c, N, S-GQDs@ZIF-8@MIP was constructed using the surface-imprinted sol–gel technique. The condensation reaction was conducted under the presence of OA, APTES, TEOS, and aqueous ammonia solution. During the polymerization process, the ultra-small size N, S-GQDs were first embedded in ZIF-8 to form N, S-GQDs@ZIF-8, and then the imprinted layer was decorated on the N, S-GQDs@ZIF-8 according to the physical deposition. After, the template molecule of OA was removed via ethanol, leaving behind 3D cavities in the imprinting layer, and obtaining a N, S-GQDs@ZIF-8@MIP sensor with selective binding sites fitting the shape of OA.

In the preparation processing of MIPs, the molar ratios of APTES and TEOS, the added quantity of supporter ZIF-8, and the amount of the fluorescent indicator N, S-GQDs had a certain impact on the imprinting factor (IF), which can directly reflect the polymer performance. Hence, to obtain materials with superior fluorescence and adsorption properties, we optimized the above aggregation conditions one by one, as presented in the supporting information file (Tables S1–S3).

### 3.2. Characterization of Materials

**Figure 1:**
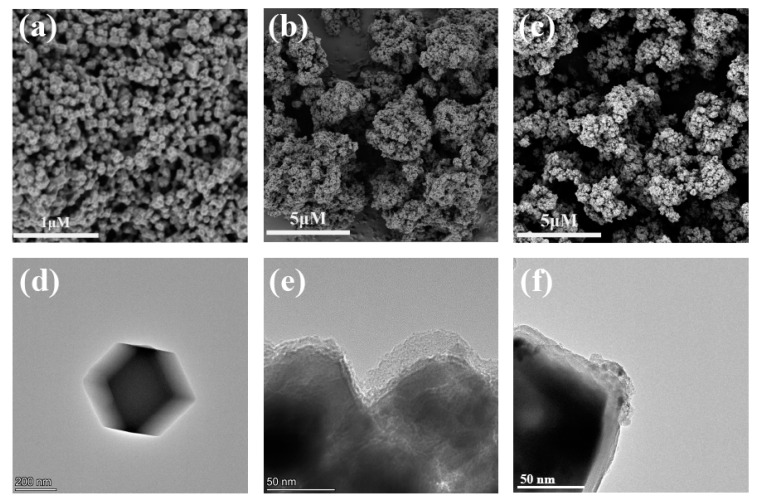
SEM images of ZIF-8 (**a**), N, S-GQDs@ZIF-8@MIP (**b**) and N, S-GQDs@ZIF-8@NIP (**c**); TEM images of ZIF-8 (**d**), N, S-GQDs@ZIF-8@MIP (**e**) and N, S-GQDs@ZIF-8@NIP (**f**).

SEM and TEM were used to observe the morphology of the obtained ZIF-8, N, S-GQDs@ZIF-8@MIP (MIP), and N, S-GQDs@ZIF-8@NIP (NIP). Figure 1a shows that the as-prepared ZIF-8 conformed to its well-known dodecahedron shape. As displayed in Figure 1d, the unmodified ZIF-8 had a smooth surface and uniform particle size. After grafting, the surface morphology of MIP (Figure 1b) and NIP (Figure 1c) presented an obvious change, and an apparent porous structure could be observed. This phenomenon indicated that the polymers were formed. TEM images of N, S-GQDS@ZIF-8 are provided in the supporting information file (Figure S1).

**Figure 2:**
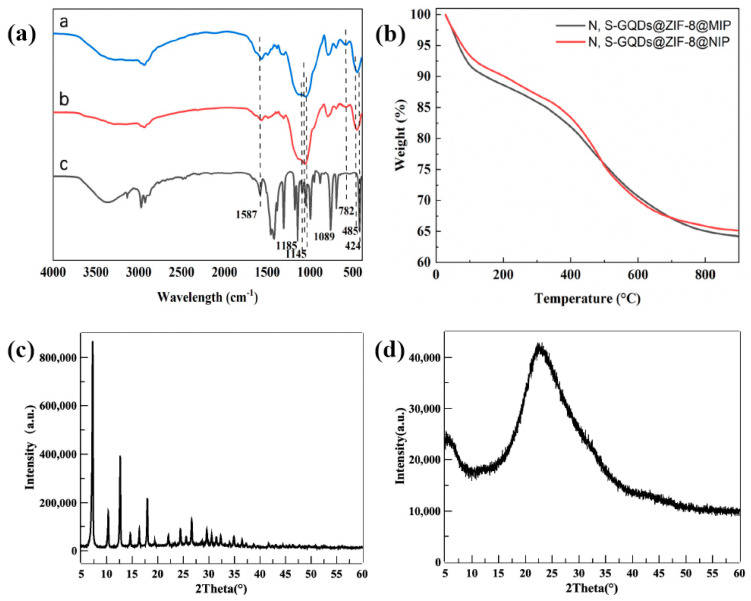
(**a**) FT-IR spectra of N, S-GQDs@ZIF-8@MIP-a, N, S-GQDs@ZIF-8@NIP-b, and ZIF-8-c. (**b**) TGA curves of N, S-GQDs@ZIF-8@MIP and N, S-GQDs@ZIF-8@NIP. XRD curves of ZIF-8 (**c**) and N, S-GQDs@ZIF-8@MIP (**d**).

The characteristics of functional groups of MIP (a), NIP (b), and ZIF-8 (c) were confirmed via FT-IR (Figure 2a). For curves a and b, characteristic peaks at 1185 cm^−1^ (C-S) and 782 cm^−1^ (C=S) can be seen in spectra a and b (Figure 2a), which indicated the successful introduction of N, S-GQDs into the composite materials [32,33]. The ZIF-8 presented characteristic absorption peaks at 1587 cm^−1^ (C=N), 1145 cm^−1^ (C-N) and 424 cm^−1^ (Zn-N bonds), as shown in Figure 2a(c). However, in curves a and b, it can be seen that the absorption peaks of ZIF-8 at 424 cm^−1^ and 1587 cm^−1^ were weakened, illustrating that the molecularly imprinted layer was successfully modified on the surface of ZIF-8. The two peaks at 1089 cm^−1^ (Si-O-Si) and 485 cm^−1^ (Si-O) indicate the existence of APTES and TEOS, respectively. The characteristic peaks in the range of 500–4000 cm^−1^ for MIP and NIP were almost identical, revealing that the template OA was removed from the imprinted polymers. The above findings indicate that MIP and NIP were successfully prepared.

TGA of MIP and NIP were performed under a stream of nitrogen. The results of weight as a function of temperature were depicted in Figure 2b. Obviously, there were three main stages of weight loss for MIP and NIP. The first stage of weight loss of MIP starting from 25 °C to 100 °C was approximately 8.50% due to the volatilization of moisture on the surface of the material and decomposition of the target that was not completely eluted. However, for NIP, weight loss was only due to volatilization of moisture on the surface of the material. Immediately afterwards, at 100–480 °C, the weight of the composite materials (MIP and NIP) was lost due to decomposition of the imprinting layer. The thermal gravimetric curves of MIP and NIP were almost the same due to the decomposition of the framework and organic polymer with the weight loss of 12.82%. The downward trends for the weight loss of MIP and NIP were similar to those of pure ZIF-8 crystals [34], demonstrating that the polymer was wrapped on the ZIF-8.

The crystal structure of synthesized ZIF-8 and MIP were analyzed using XRD. The pattern of ZIF-8 shown in Figure 2c matched well with the simulated results for the reported single-crystal data in the literature [35], revealing the successful synthesis of ZIF-8. The synthesized MIP showed the XRD pattern illustrated in Figure 2d. After polymerization, the diffraction peaks of ZIF-8 almost disappeared, which indicated the successful coating of MIP. Meanwhile, a new broad characteristic vibration peak located at 2θ = 23.5° appeared in MIP (Figure 2d), which stemmed from the introduction of MIPs and N, S-GQDs to the (002) graphite planes.

### 3.3. Photoluminescence Properties and Kinetic Absorption of N, S-GQDs@ZIF-8@MIP 

**Figure 3:**
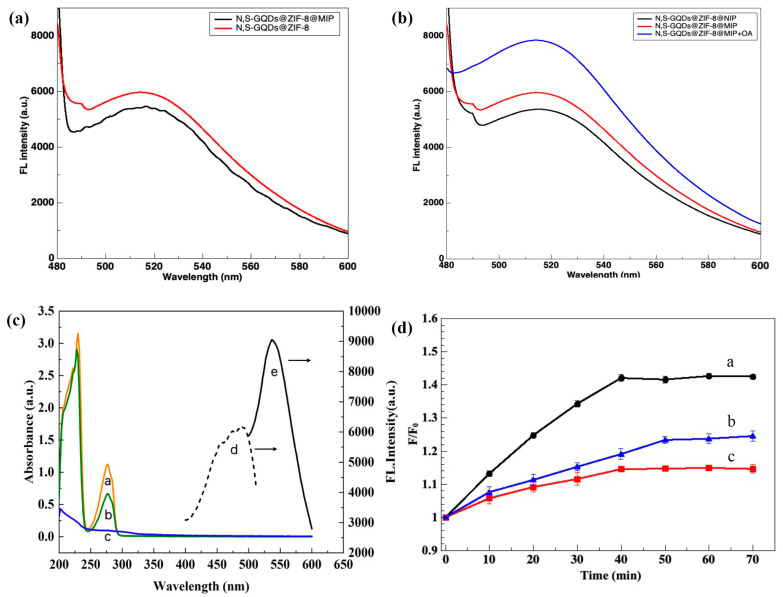
(**a**) Fluorescence spectra N, S-GQDs@ZIF-8 and N, S-GQDs@ZIF-8@MIP under 460 nm excitation. The concentrations of N, S-GQDs@ZIF-8 and N, S-GQDs@ZIF-8@MIP were 1 mg mL^−1^. (**b**) Fluorescence emission spectra of N, S-GQDs@ZIF-8@MIP, N, S-GQDs@ZIF-8@NIP and N, S-GQDs@ZIF-8@MIP with OA. (**c**) UV-Vis absorption spectra of OA-a, N, S-GQDs@ZIF-8@MIP before-c and after-b interactions with OA; fluorescence excitation-d, and emission-e spectra of the N, S-GQDs@ZIF-8@MIP. (**d**) Fluorescence response of N, S-GQDs@ZIF-8@MIP (a), N, S-GQDs@MIP (b) and N, S-GQDs@ZIF-8@NIP (c) under different contact times.

The optical properties of N, S-GQDS@ZIF-8@MIP were also investigated. As illustrated in Figure 3a, N, S-GQDS@ZIF-8@MIP exhibited symmetrical fluorescence emissions at 520 nm under excitation at 460 nm. The FL intensity of N, S-GQDS@ZIF-8@MIP was effectively enhanced when OA molecules were included in the imprinted cavities (Figure 3b). The FL intensity of N, S-GQDS@ZIF-8@MIP was recovered to 109.5% of that of N, S-GQDS@ZIF-8@NIP when removing the target molecules with ethanol as the elution solution.

Under the presence of octopamine, N, S-GQDs@ZIF-8@MIP revealed fluorescence enhancement. For this phenomenon, a fluorescence enhancement mechanism was proposed to explain the interaction between octopamine and N, S-GQDs@ZIF-8@MIP. First, as displayed in Figure 3c, the UV absorption spectrum of octopamine (a) did not overlap with the emission peak for N, S-GQDs@ZIF-8@MIP(e) of the fluorophore, which eliminated the possibility of fluorescence resonance energy transfer (FRET). Octopamine is a well-known aromatic compound with a large π bond benzene ring that can easily generate photoelectrons. Meanwhile, octopamine also has common electron-donating groups with amino and hydroxyl groups that can help the electron transfer from octopamine to fluorescent substances. Thus, the mechanism of fluorescence enhancement was speculated as a process that mostly relies on the electron transfer from octopamine to N, S-GQDs@ZIF-8@MIP. To further confirm this structure, the fluorescence lifetime of the materials was measured using time-resolved spectroscopy, which was used to monitor the emission of the 520 nm fluorophore in the absence and presence of the analyte. The results are provided in the supporting information file (Figure S2). According to the above analysis, the main mechanism for the fluorescence enhancement of N, S-GQDs@ZIF-8@MIP may be photo-induced electron transfer (PET).

To investigate the absorption ability of N, S-GQDs@ZIF-8@MIP to OA, experiments were designed using N, S-GQDs@ZIF-8@NIP and N, S-GQDs@MIP as comparisons. 

As illustrated in Figure 3d, the absorption kinetics of N, S-GQDs@ZIF-8@MIP (a), N, S-GQDs@ZIF-8@NIP (b), and N, S-GQDs@MIP (c) were investigated by determining the variation in the fluorescence response (*F/F*_0_) from 0 to 70 min. With a change in the adsorption time, the F/F_0_ showed a remarkable increase. The adsorption of OA by N, S-GQDs@ZIF-8@MIP occurred in the first 30 min, and then slowly increased between 30 min and 40 min. After 40 min, there was no noticeable alteration. Similar absorption trends were observed for N, S-GQDs@ZIF-8@NIP. However, N, S-GQDs@ZIF-8@MIP displayed a higher *F/F*_0_ value than N, S-GQDs@ZIF-8@NIP under the same conditions, which may be ascribed to the specific binding between OA and the -OH/-NH_2_ groups of the imprinting recognition sites on the N, S-GQDs@ZIF-8@MIP. Meanwhile, the absorption performance of N, S-GQDs@ZIF-8@MIP was better than that of N, S-GQDs@MIP. Additionally, compared to N, S-GQDs@MIP, the adsorption equilibrium time of N, S-GQDs@ZIF-8@MIP was greatly shortened from 50 min to 40 min. These results indicated that the introduction of high surface area ZIF-8 can provide a platform for the MIP membrane, which can allow more imprinted sites on the surface and enhance the adsorption capacity of materials. The excellent OA adsorption by N, S-GQDs@ZIF-8@MIP illustrated that the introduction of high-surface-area ZIF-8 indeed improved the adsorption competence of the sensor.

### 3.4. Establishment of Fluorescence Sensing Detection for OA

**Figure 4:**
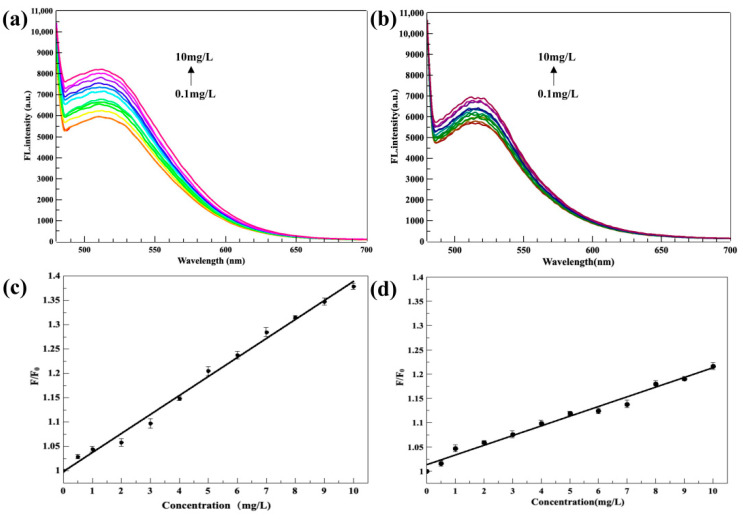
Fluorescence emission spectra of N, S-GQDs@ZIF-8@MIP (**a**) and N, S-GQDs@ZIF-8@NIP (**b**) under the different OA concentrations, the linear section of N, S-GQDs@ZIF-8@MIP (**c**) and N, S-GQDs@ZIF-8@NIP (**d**).

To evaluate the dose–response curves of the prepared fluorescent materials for OA, N, S-GQDs@ZIF-8@MIP and N, S-GQDs@ZIF-8@NIP were dispersed in OA ethanol solution with various concentrations from 0.1 to 10 mg L^−1^. The fluorescence intensity of the polymers gradually increased with an increase in the concentration of OA, and the results are shown in Figure 4. Compared to N, S-GQDs@ZIF-8@NIP, N, S-GQDs@ZIF-8@MIP produced a greater enhancement effect at the same OA concentration. The relationship between the fluorescence response of the sensor and the concentration of the OA conformed to the following equation [36]:

*F*/*F*_0_= 1 + *K_SV_C_OA_*

where *F*_0_ and *F* are the fluorescence intensity in the absence and presence of OA, respectively; *K_SV_* represents the constant; and *C_OA_* is the concentration of OA. Herein, the imprinting factor IF (*K_SV, N, S-GQDS@ZIF-8@MIP_/K_SV, N, S-GQDS@ZIF-8@NIP_*) was used as an important indicator for evaluating the binding ability of the N, S-GQDs@ZIF-8@MIP sensor.

As expected, the fluorescence response sharply increased along with an increase in OA concentration (0.1–10 mg L^−1^), which can be seen in Figure 4a. The corresponding equation with a good linear relationship of *F/F*_0_ = 0.039 *C_OA_* + 0.9984 was obtained for N, S-GQDs@ZIF-8@MIP with correlation coefficients of 0.9933. The limit of detection (LOD) was determined to be 0.062 mg L^−1^, and was calculated following the standard 3σ criteria (3*σ/S*) [37]. As shown in Figure 4b, the corresponding standard linear equation of N, S-GQDs@ZIF-8@NIP was *F/F*_0_ = 0.02 *C_OA_* + 1.013 with correlation coefficients of 0.983. As displayed in Figure 4, under identical conditions, the N, S-GQDs@ZIF-8@MIP exhibited greater fluorescence enhancement than N, S-GQDs@ZIF-8@NIP due to the absence of OA-imprinted cavities in N, S-GQDs@ZIF-8@NIP. Under optimal conditions, the calculated *IF* was 1.95, which indicated that the constructed N, S-GQDs@ZIF-8@MIP had an excellent affinity for OA.

### 3.5. Selectivity of N, S-GQDs@ZIF-8@MIP

**Figure 5:**
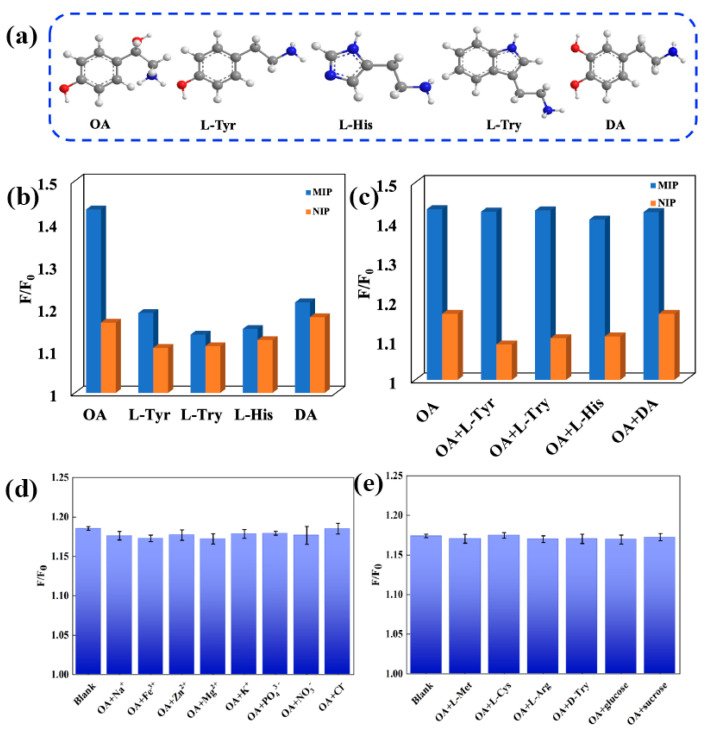
(**a**) The structure of OA, L-Try, L-Tyr, L-His, and DA. (**b**) Changes in the fluorescence intensity of N, S-GQDs@ZIF-8@MIP and N, S-GQDs@ZIF-8@NIP in response to OA and responses to the other analogs. (**c**) The effect of analogues on the binding of OA to the N, S-GQDs@ZIF-8@MIP and N, S-GQDs@ZIF-8@NIP. Influence of common coexisting ions (**d**) and other molecules (**e**) on the increasing fluorescence efficiency of N, S-GQDs@ZIF-8@MIP in the presence of OA.

To investigate the selectivity of N, S-GQDs@ZIF-8@MIP(MIP) towards OA, a series of experiments was carried out using L-Try, L-Tyr, L-His, and DA as the structural analogues of OA. The structures of the analogues and OA are provided in Figure 5a. Additionally, the fluorescence responses (*F/F*_0_) of MIP and N, S-GQDs@ZIF-8@NIP(NIP) to OA, L-Try, L-Tyr, and L-His are presented in Figure 5b. MIP clearly had higher adsorption capabilities for OA in the selectivity experiments and displayed little sensitivity to non-template molecules (L-Try, L-Tyr, L-His, and DA). The fluorescence response of NIP to OA was as weak as that of other structural analogues (L-Try, L-Tyr, L-His, and DA) because of the lack of specific imprinting sites.

Competitive adsorption experiments were also conducted by adding different structural analogues (L-Try, L-Tyr, L-His, and DA) into the solution containing OA. The fluorescence responses (*F/F*_0_) of MIP and NIP were enhanced with almost the same efficiency (Figure 5c). The above results confirmed that MIP had excellent selectivity towards OA. On the basis of these observations, we can draw the following conclusion: MIP with imprinted cavities has a stronger affinity for OA.

Furthermore, some substances introduced by the sample pretreatments, including common metal ions (Na^+^, K^+^, Mg^2+^, Fe^3+^, and Zn^2+^), amino acids (D-Try, L-Arg, L-Met, and L-Cys), and sugars (glucose and sucrose), may have affected the accuracy of the experimental results. Based on the presence of these interfering substances, an experiment was designed to confirm the specificity of the synthesized sensor in complex environments. We determined that even at concentrations up to 1 g L^−1^ (250-fold that of OA), these substances caused only a slight fluorescence change of MIP (Figure 5d,e).

These experiments confirmed that the obtained sensor has high sensitivity and excellent anti-interference capability for the recognition of OA. Thus, we conclude that the constructed MIP was a well-fluorescent material that can effectively detect OA in fermented samples.

### 3.6. OA Detection in Real Samples

To further evaluate the accuracy and applicability of the constructed N, S-GQDs@ZIF-8@MIP, a comparison between the constructed fluorescence-sensing method and classical detection method (HPLC-UV) was carried out. In this work, the actual samples were white vinegar and spirits. Since OA was not found in the two blank samples using the FL and HPLC methods, the recovery experiments were performed by spiking different concentrations of OA (1, 5, and 9 mg L^−1^), and three parallel groups were set for the determination of each concentration of each sample. As outlined in Table 1, the relative recovery ratio and the relative standard deviation (RSD) of the sample solutions were determined under the condition that the same concentration was repeated three times, which was calculated from the following equation: relative recovery (%) = (C_F_ − C_S_/C_A_) × 100, where C_F_ is the total OA found in a spiked sample, C_S_ is the OA in the original sample and C_A_ is the standard OA spiked in sample. In real sample detection, the FL method recoveries of OA were between 80.24% and 98.11%, and the RSD was in the range of 2.7–5.2. In addition, the HPLC method was used to verify the reliability of the developed FL test results, and the recoveries ranged from 80.38% to 87.17%, while RSD was in the range of 1.22–4.93. The results displayed that there was no significant difference (*p* value = 0.96 > 0.05) between this method and HPLC. By comparing the detection results under FL with those of HPLC, we determined that the N, S-GQDs@ZIF-8@MIP-based sensor can supply a well-pleasing platform for determination of OA in samples.

### 3.7. Method Performance Comparison

The performance of N, S-GQDs@ZIF-8@MIP-based fluorescence-sensing method was compared with the previous reported approaches for OA detection in Table 2. Apparently, the FL method constructed in this work displayed a lower detection limit, good precision (RSD) and linear range. The excellent performance of N, S-GQDs@ZIF-8@MIP-based sensing system benefited from the following aspects. Firstly, N, S-GQDs@ZIF-8 with extremely bright green light and large surface area was introduced as supporter, which can not only evidently improve the sensitivity of the sensor, but also reduced the aggregation quenching of N, S-GQDs. Secondly, the modification of the imprinting layer further enhanced the accuracy of the sensor in identifying target molecules, which was beneficial to avoiding tedious sample pre-processing and shortening analysis time. In summary, apart from the distinct advantages of specific recognition sites, the presented method was also simple in operation, rapid in response and cost-effective. Thence, the developed fluorescence imprinting sensor was more suitable and promising for the specific recognition and quantitative detection of trace OA in the fermented environment.

## 4. Conclusions

In this paper, an efficient one-pot strategy was proposed to construct a novel N, S-GQDs@ZIF-8@MIP sensor that can achieve OA detection through photo-induced electron transfer. Through the introduction of N, S-GQDs with unique fluorescence properties, the obtained N, S-GQDs@ZIF-8@MIP presented a more significant fluorescence response to trace OA. The modification of molecular imprinting with the special recognition sites gave the sensor good selectivity. Meanwhile, the use of ZIF-8 with a high specific surface area improved the mass transfer rate of the N, S-GQDs@ZIF-8@MIP. The synthesized N, S-GQDs@ZIF-8@MIP sensor has the merits of favorable selectivity and higher sensitivity. More importantly, N, S-GQDs@ZIF-8@MIP was successfully applied to the detection of OA in real samples. Therefore, N, S-GQDs@ZIF-8@MIP has a promising prospect for OA detection in fermented foods.

## Figures and Tables

**Table 1:** The determination of OA-containing samples via the proposed N, S-GQDs@ZIF-8@MIP and HPLC-UV.

Samples	Added (mg L^−1^)	N, S-GQDs@ZIF-8@MIP			HPLC-UV		
		Found (mg L^−1^) (n = 3)	Recovery% (Mean ± SD) (n = 3)	RSD% (n = 3)	Found (mg L^−1^) (n = 3)	Recovery % (Mean ± SD) (n = 3)	RSD% (n = 3)
white vinegar	0.00	ND ^a^	-	-	ND ^a^	-	-
	1.00	0.87	86.86 ± 2.31	2.66	0.80	80.38 ± 3.96	4.93
	5.00	4.68	93.58 ± 1.64	1.75	4.30	85.90 ± 1.04	1.22
	9.00	8.00	88.92 ± 2.46	2.77	7.27	80.75 ± 2.60	3.23
wine	0.00	ND ^a^	-	-	ND ^a^	-	-
	1.00	0.91	91.08 ± 1.68	1.84	0.84	84.48 ± 2.24	2.65
	5.00	4.54	90.90 ± 4.13	4.54	4.36	87.17 ± 1.81	2.09
	9.00	8.29	92.07 ± 2.66	2.89	7.41	82.31 ± 2.36	2.87

^a^ Not Detectable.

**Table 2:** Comparison of the proposed method with other methods reported in the literature.

Methods	Detector	LOD (mg L^−1^)	Linear Range (mg L^−1^)	Recovery (%)	RSD (%)	Advantages	Limitations	Reference
Electrochemical	octopamine tyramine	0.153 0.041	0.0153–6.12 0.013–0.034	98.5–104.7 102.2–103.1	5.9–6.1 5.6–6.4	fast sensitive	The scope of use is limited	[5]
ELISA	octopamine	0.59	2.259–15.3	96.2–106.4	-	high reproducibility	Low sensitivity and specificity	[6]
FSCV	octopamine	0.336	-	-	-	sensitive	Low accuracy, easily disturbed	[7]
HPLC	synephrine octopamine	0.033 0.092	0.2–1.2 0.2–1.2	98.2–101.5	1.5–2.5	wide range of applications	Costly, complex preprocessing	[8]
Fluorescence (UCNPs@ZIF-8@MIP)	octopamine	0.081	0.1–10	81.75–90.63	2.56–5.13	simple preprocessing	UCNP synthesis conditions were complex	[28]
Fluorescence (N,S-GQDs@ZIF-8@MIP)	octopamine	0.062	0.1–10	86.86–93.58	1.75–4.54	fast, sensitive, high detection limits		This work

## Data Availability

The datasets generated for this study are available on request from the corresponding author.

## Supplementary Materials

The following are available online at https://www.mdpi.com/article/10.3390/foods11091348/s1, Figure S1: The high-angle annular dark-field (HAADF) image of N, S-GQDs@ZIF-8, and corresponding element mappings of C (B), N (C), O (D), S (E), and Zn (F); Figure S2: Fluorescence emission decay curves of N, S-GQDs@ZIF-8@MIP alone and N, S-GQDs@ZIF-8@MIP with 40 mg mL^−1^ concentrations of OA under excitation at 460 nm. The concentration of the N, S-GQDs@ZIF-8@MIP was 2 mg mL^−1^, Figure S3: The stability of N, S-GQDs@ZIF-8@MIP from 0 to 720 min at room temperature; Table S1: Optimization of addition ratio; Table S2: Optimization of ZIF-8; Table S3: Optimization of N, S-GQDs dosage.

## References

[1] Tofalo R., Perpetuini G., Schirone M., Suzzi G. (2015). Biogenic Amines: Toxicology and Health Effect. Encycl. Food Health.

[2] Elias D.O., Land B.R., Mason A.C., Hoy R.R. (2006). Measuring and quantifying dynamic visual signals in jumping spiders. J. Comp. Physiol. A Sens. Neural Behav. Physiol..

[3] Wójcik W., Łukasiewicz M., Puppel K. (2020). Biogenic amines: Formation, action and toxicity—A review. J. Sci. Food Agric..

[4] Thevis M., Koch A., Sigmund G., Thomas A., Schänzer W. (2011). Analysis of octopamine in human doping control samples. Biomed. Chromatogr..

[5] Zhang Y., Zhang M., Wei Q., Gao Y., Guo L., Al-Ghanim K.A., Mahboob S., Zhang X. (2016). An Easily Fabricated Electrochemical Sensor Based on a Graphene-Modified Glassy Carbon Electrode for Determination of Octopamine and Tyramine. Sensors.

[6] Moczko E., Díaz R., Rivas B., García C., Pereira E., Piletsky S., Cáceres C. (2019). Molecularly Imprinted Nanoparticles Assay (MINA) in Pseudo ELISA: An Alternative to Detect and Quantify Octopamine in Water and Human Urine Samples. Polymers.

[7] Pyakurel P., Champaloux E.P., Venton B.J. (2016). Fast-Scan Cyclic Voltammetry (FSCV) Detection of Endogenous Octopamine in Drosophila melanogaster Ventral Nerve Cord. ACS Chem. Neurosci..

[8] Pawar R.S., Sagi S., Leontyev D. (2020). Analysis of bitter orange dietary supplements for natural and synthetic phenethylamines by LC–MS/MS. Drug Test. Anal..

[9] Kang J.H., Kim C. (2018). Colorimetric detection of iron and fluorescence detection of zinc and cadmium by a chemosensor containing a bio-friendly octopamine. Photochem. Photobiol. Sci..

[10] Gupta V., Chaudhary N., Srivastava R., Sharma G.D., Bhardwaj R., Chand S. (2011). Luminscent Graphene Quantum Dots for Organic Photovoltaic Devices. J. Am. Chem. Soc..

[11] Du Y., Guo S. (2015). Chemically doped fluorescent carbon and graphene quantum dots for bioimaging, sensor, catalytic and photoelectronic applications. Nanoscale.

[12] Kundu S., Yadav R.M., Narayanan T.N., Shelke M.V., Vajtai R., Ajayan P.M., Pillai V.K. (2015). Synthesis of N, F and S Co-Doped Graphene Quantum Dots. Nanoscale.

[13] Qu D., Zheng M., Zhang L., Zhao H., Xie Z., Jing X., Haddad R.E., Fan H., Sun Z. (2014). Formation mechanism and optimization of highly luminescent N-doped graphene quantum dots. Sci. Rep..

[14] Li X., Lau S.P., Tang L., Ji R., Yang P. (2014). Sulphur doping: A facile approach to tune the electronic structure and optical properties of graphene quantum dots. Nanoscale.

[15] Li S., Li Y., Cao J., Zhu J., Fan L., Li X. (2014). Sulfur-Doped Graphene Quantum Dots as a Novel Fluorescent Probe for Highly Selective and Sensitive Detection of Fe^3+^. Anal. Chem..

[16] Xia C., Hai X., Chen X.-W., Wang J.-H. (2017). Simultaneously fabrication of free and solidified N, S-doped graphene quantum dots via a facile solvent-free synthesis route for fluorescent detection. Talanta.

[17] Yaghi O.M., Li G., Li H. (1995). Selective binding and removal of guests in a microporous metal–organic framework. Nature.

[18] Pham T., Forrest K.A., Franz D.M., Space B. (2017). Experimental and theoretical investigations of the gas adsorption sites in rht-metal–organic frameworks. CrystEngComm.

[19] Li J., Wang X., Zhao G., Chen C., Chai Z., Alsaedi A., Hayat T., Wang X. (2018). Metal–organic framework-based materials: Superior adsorbents for the capture of toxic and radioactive metal ions. Chem. Soc. Rev..

[20] Altintas C., Avci G., Daglar H., Azar A.N.V., Velioglu S., Erucar I., Keskin S. (2018). Database for CO_2_ Separation Performances of MOFs Based on Computational Materials Screening. ACS Appl. Mater. Interfaces.

[21] Zhou Y., Li X., Pan Z., Ye B., Xu M. (2019). Determination of Malachite Green in Fish by a Modified MOF-Based Electrochemical Sensor. Food Anal. Methods.

[22] Zhu Q.-L., Xu Q. (2014). Metal-organic framework composites. Chem. Soc. Rev..

[23] Tripathy S.P., Subudhi S., Parida K. (2021). Inter-MOF hybrid (IMOFH): A concise analysis on emerging core–shell based hierarchical and multifunctional nanoporous materials. Coord. Chem. Rev..

[24] Xu L., Fang G., Liu J., Pan M., Wang R., Wang S. (2016). One-pot synthesis of nanoscale carbon dots-embedded metal–organic frameworks at room temperature for enhanced chemical sensing. J. Mater. Chem. A.

[25] Chen L., Wang X., Lu W., Wu X., Li J. (2016). Molecular imprinting: Perspectives and applications. Chem. Soc. Rev..

[26] Malik M.I., Shaikh H., Mustafa G., Bhanger M.I. (2018). Recent Applications of Molecularly Imprinted Polymers in Analytical Chemistry. Sep. Purif. Rev..

[27] Rutkowska M., Płotka-Wasylka J., Morrison C., Wieczorek P.P., Namiesnik J., Marć M. (2018). Application of molecularly imprinted polymers in analytical chiral separations and analysis. TrAC Trends Anal. Chem..

[28] Caoa Y., Hua X., Zhaoa T., Maoa Y., Fanga G., Wangab S. (2020). A core-shell molecularly imprinted optical sensor based on the upconversion nanoparticles decorated with Zinc-based metal-organic framework for selective and rapid detection of octopamine. Sensors Actuators B Chem..

[29] Hu X., Cao Y., Tian Y., Qi Y., Fang G., Wang S. (2020). A molecularly imprinted fluorescence nanosensor based on upconversion metal–organic frameworks for alpha-cypermethrin specific recognition. Mikrochim. Acta.

[30] Qu D., Sun Z., Zheng M., Li J., Zhang Y., Zhang G., Zhao H., Liu X., Xie Z. (2015). Three Colors Emission from S.N. Co-doped Graphene Quantum Dots for Visible Light H_2_Production and Bioimaging. Adv. Opt. Mater..

[31] Chen S., Hai X., Xia C., Chen X.-W., Wang J.-H. (2013). Preparation of Excitation-Independent Photoluminescent Graphene Quantum Dots with Visible-Light Excitation/Emission for Cell Imaging. Chem. A Eur. J..

[32] Qu D., Zheng M., Du P., Zhou Y., Zhang L., Li D., Tan H., Zhao Z., Xie Z., Sun Z. (2013). Highly luminescent S, N co-doped graphene quantum dots with broad visible absorption bands for visible light photocatalysts. Nanoscale.

[33] Xu H., Zhou S., Xiao L., Yuan Q., Gan W. (2016). Time-efficient syntheses of nitrogen and sulfur co-doped graphene quantum dots with tunable luminescence and their sensing applications. RSC Adv..

[34] Cravillon J., Münzer S., Lohmeier S.-J., Feldhoff A., Huber K., Wiebcke M. (2009). Rapid Room-Temperature Synthesis and Characterization of Nanocrystals of a Prototypical Zeolitic Imidazolate Framework. Chem. Mater..

[35] He L., Wang T., An J., Li X., Zhang L., Li L., Li G., Wu X., Su Z., Wang C. (2014). Carbon nanodots@zeolitic imidazolate framework-8 nanoparticles for simultaneous pH-responsive drug delivery and fluorescence imaging. CrystEngComm.

[36] Yang J., Feng W., Liang K., Chen C., Cai C. (2020). A novel fluorescence molecularly imprinted sensor for Japanese encephalitis virus detection based on metal organic frameworks and passivation-enhanced selectivity. Talanta.

[37] (1987). Analytical Methods Committee Recommendations for the definition, estimation and use of the detection limit. Analyst.

