# Supplementary material for: A High-Luminescence Biomimetic Nanosensor Based on N, S-GQDs-Embedded Zinc-Based Metal–Organic Framework@Molecularly Imprinted Polymer for Sensitive Detection of Octopamine in Fermented Foods
Source: Foods. 2022 May 6;11(9):1348. doi: 10.3390/foods11091348 (PMC9100785; doi:10.3390/foods11091348)
Supplement: Supplementary file 1 [file foods-11-01348-s001.zip › foods-1616046-supplementary.pdf]

Supplementary Materials

Contents

Supplementary experimental method

Table S1 Table S2 Table S3

Figure S1 Figure S2 Figure S3

Instruments

Fluorescence spectra were performed on an F-7100 fluorescence spectrophotometer(Thermo, USA, http://www.hitachi.com) at the excitation wavelength of 460 nm. Fourier transform infrared (FT-IR) were performed to analyze the obtained materials on a Tensor-37 FTIR spectrophotometer (Bruker, Germany, https://www.bruker.com). Scanning electron microscope (SEM, LEO, Germany) and transmission electron microscope (TEM, JEOL, Japan, https://www.jeol.co.jp) images were used to show the surface and structure of the obtained materials. Ultraviolet-visible (UV-Vis) absorption spectra were obtained on UV-Cary100 spectrophotometer (Shimadzu, Japan). Thermogravimetric analysis (TGA) was carried out with a PTC-10 A thermal gravimetric analyzer (Rigaku Corp, https://www.instrument.com.cn) from room temperature to 900°C. X-ray diffraction (XRD) data was measured at the angular range of 5-60 degrees (2θ) with Cu Kα radiation.

Detailed Pre-treatment of samples

For HPLC detection, the samples that were extracted as abovementioned were derivatized with dansyl chloride and then analysed according to the National Standards

of the People's Republic of China (GB 5009.208-2016). The determination was performed by HPLC system with ultraviolet detector. The separation was carried out with a reversed phase column (C18, 250 mm × 4.6 mm, 5 μm particle size) with a flow rate of 0.8 mL min−1 and column temperature of 35 °C. The injection volume was 20 μL, and the detection wavelength was 254 nm.

Optimization the conditions of N, S-GQDs@ZIF-8@MIP

In the preparation processing of molecularly imprinted polymers, the molar ratios of APTES and TEOS, the amount of support carrier ZIF-8, and the amount of the fluorescent indicator N, S-GQDs had a certain effect the imprinting factor (*IF*) that can directly reflect the polymer performance. Therefore, we optimized the above aggregation conditions one by one.

As we all know, the molar ratio both functional monomer and cross-linker played a critical role in the performance of synthetic molecular imprinting polymers. As shown in Table S1, the imprinting factor of N, S-GQDs@ZIF-8@MIP towards OA increased from 1.40 to 2.71 with the increase of the molar proportion ranging from 1: 4: 4 to 1: 4: 6. Then, IF slightly decreased to 1.28 with the molar ration increased to 1: 4: 8. The above results indicated that the low molar ratio was not conducive to the formation of a good network structure of the polymer, which led to a low adsorption efficiency of N, S-GQDs@ZIF-8@MIP towards OA. Meanwhile, excessive amount of cross-linker caused an increase of non-specific adsorption sites, which in turn reduced the specificity recognition of polymer materials for template. Thus, 1:4:6 was determined

as the best addition ratio.

Additionally, the support carrier ZIF-8 also acted as a vital role in the polymerization reaction. As shown in Table S2, as the dosage of ZIF-8 increased from 12.5 mg to 50 mg, the imprinting factor first increased significantly from 2.21 to 4.57 and then decreased to 2.77. According to the result of Table S2, the best addition of ZIF-8 was 25 mg.

As fluorescent indicator, the amount of N, S-GQDs was directly related to the fluorescent performance of the synthetic material and the sensitivity of the sensing dsystem. As displayed in Table S3, the imprinting factor (*IF*) increased from 3.43 to 4.28 with the initial addition of N, S-GQDs from 50 μL to 100 μL. However, when the amount of N, S-GQDs is 300 μL, the *IF* decreased to 1.82. When the addition amount of N, S-GQDs was insufficient, the synthetic material exhibited low fluorescence intensity, and the fluorescence response of the synthetic material to the template molecule was reduced, which cannot meet the needs of subsequent detection. Similarly, excessive N, S-GQDs will cause the reduction of the sensitivity of sensor. As a compromise to acquire the superior fluorescent performance, the best N, S-GQDs addition was100 μL.

Table S1: Optimization of addition ratio.

Polymer	Ratio	F/F_0 (MIP)	F/F_0 (NIP)	*IF*
MIP_1	1:4:4	1.1151	1.0820	1.40
MIP_2	1:4:6	1.0930	1.0343	2.71
MIP_3	1:4:10	1.1200	1.0938	1.28

Table S2: Optimization of ZIF-8 dosage.

Polymer	Dosage (mg)	F/F_0 (MIP)	F/F_0 (NIP)	*IF*
MIP_1	12.5	1.0987	1.0447	2.21
MIP_2	25	1.1566	1.0343	4.57
MIP_3	50	1.0587	1.0212	2.77

Table S3: Optimization of N, S-GQDs dosage.

Polymer	Dosage (μ l)	F/F_0 (MIP)	F/F_0 (NIP)	*IF*
MIP_1	50	1.264	1.077	3.43
MIP_2	100	1.568	1.133	4.28
MIP_3	300	1.405	1.223	1.82

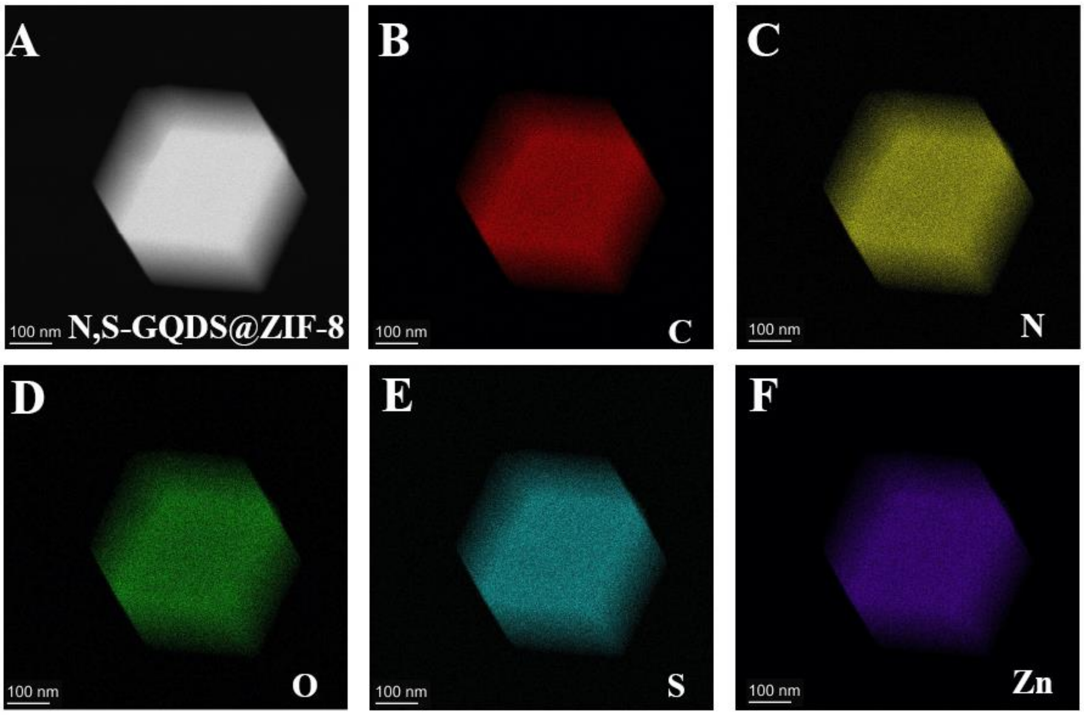

Figure S1: The high-angle annular dark-field (HAADF) image of N, S-GQDs@ZIF-8, and corresponding element mappings of C (B), N (C), O (D), S (E) and Zn (F).

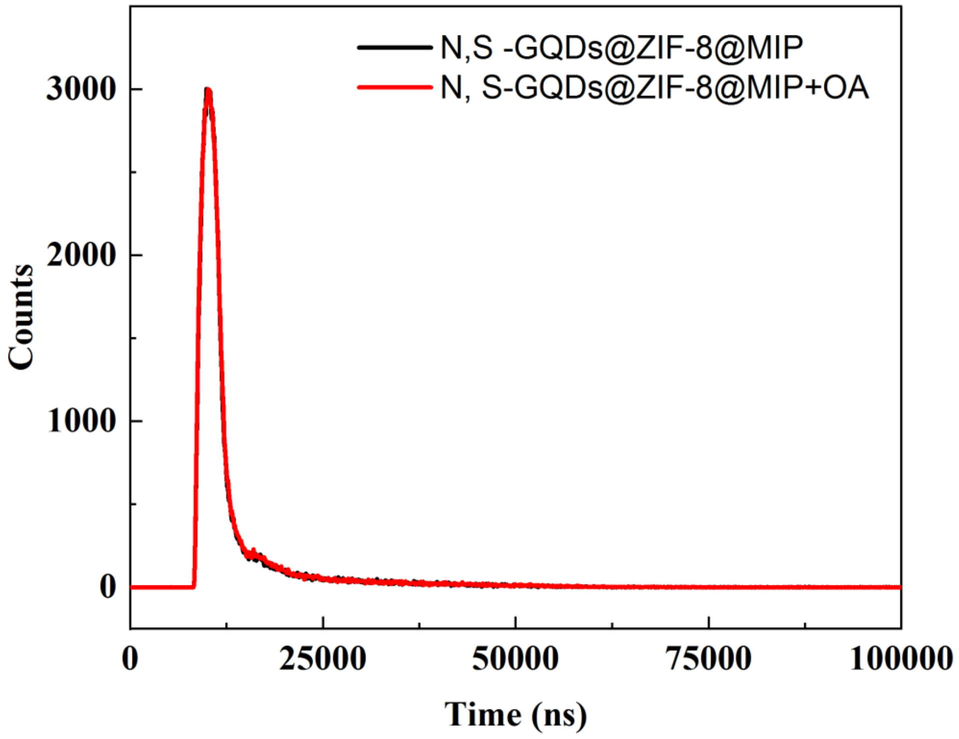

Figure S2: Fluorescence emission decay curves of N, S-GQDs@ZIF-8@MIP alone and N, S-GQDs@ZIF-8@MIP with 40 mg mL^{-1} concentrations of OA under excitation at 460 nm. The concentration of the N, S-GQDs@ZIF-8@MIP was 2 mg mL^{-1}.

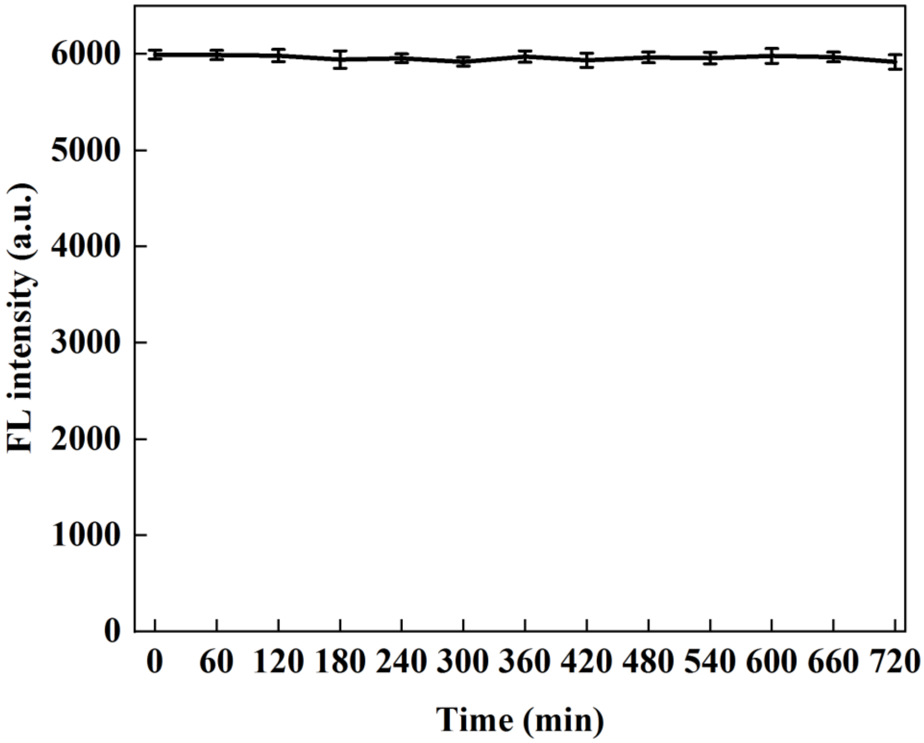

Figure S3: The stability of N, S-GQDs@ZIF-8@MIP from 0 to 720 min at room temperature.
